# Diversities in the place of delivery choice: a study among expectant mothers in Ghana

**DOI:** 10.1186/s12884-022-05158-0

**Published:** 2022-11-25

**Authors:** Mahama Ibrahim Baba, Kofi Adesi Kyei, Justina Baaba Kyei, Joseph Daniels, Isaac Justice Kobina Biney, John Oswald, Patrick Tschida, Michael Brunet

**Affiliations:** 1 Department of Public Health, Tamale Teaching Hospital, Tamale, Ghana; 2 School of Biomedical and Allied Health Sciences, University of Ghana, Accra, Ghana; 3 Korle-Bu Teaching Hospital, Accra, Ghana; 4 Public Health Directorate, Tema West, Ghana; 5 Britannia Medical Centre, Tema, Ghana; 6 Faculty of Public Health, Walden University, Minneapolis, USA

**Keywords:** Delivery, Expectant mothers, Childbirth, Culture

## Abstract

**Background:**

In this study, the factors that influence the choice of place of delivery among expectant mothers in both rural and urban settings in the northern part of Ghana were identified and compared using the conceptual framework provided by Thaddeus and Maine.

**Methods:**

A mixed-method study was used to examine expectant mothers and their responses related to factors that affect their choice of place of delivery through a concurrent triangulation using health professional interviews and a detailed participant survey. The sample consisted of 552 expectant mothers between the ages of 15 and 49 years. Individual interviews were conducted with 8 health professionals. There was also a focus group discussion with randomly selected pregnant women and lactating mothers. Themes were generated through open coding of the interview data, while multiple regression was performed to identify the factors associated with choice of place of delivery.

**Results:**

Major preference (60.1%) was for home delivery among rural dwellers compared to 20.7% for urban participants. Statistically significant variables affecting the choice of place of delivery among study participants were found to be educational background, the experience of previous deliveries, the attitude of hospital staff toward pregnant women during labor, and frequency of accessing antenatal care.

**Conclusion:**

Majority of rural women prefer home delivery to facility delivery which is the opposite of the trend observed among urban women. The study’s implications may lead to positive change where stakeholders develop and implement policies to promote health facility delivery for expectant mothers in Ghana.

**Supplementary Information:**

The online version contains supplementary material available at 10.1186/s12884-022-05158-0.

## Background

The World Health Organization (WHO), the United Nations Children’s Fund (UNICEF), the United Nations Population Fund (UNFPA), and the World Bank [1] define maternal mortality (MM) as “female deaths due to any cause associated with or worsened by pregnancy or its management (excluding accidental or incidental causes) occurring during pregnancy and childbirth or within 42 days of termination of a pregnancy, irrespective of the duration and site of the pregnancy”. The chances of mothers in Ghana surviving childbirth may either increase or decrease depending on where they deliver their babies. Over the years, indicators for maternal mortality have remained relatively high for much of sub-Saharan Africa [2]. The Ghana Maternal Health Survey (GMHS) conducted in 2017 estimated maternal mortality at 310 deaths per 100,000 live births [3]. In 2015, Ghana failed to attain the target of a 75% reduction in MM set as part of the Millennium Development Goals (MDGs) [4] and is currently behind schedule on target 3.1 of the Sustainable development Goals (SDGs) which aims at achieving global MM lower than 70 per 100,000 live births by 2030 [5]. According to Birmeta et al. [6] the main cause of high rates of maternal deaths is the lack of access to health services during pregnancy and at delivery. This problem is more pronounced in developing countries and may partially explain the disparity in mortality rates between developing and developed countries.

In Ghana, the place of delivery has a direct effect on the outcome of delivery and MM. Expectant mothers have the option to deliver either at a health institution with trained personnel or at home with the assistance of traditional birth attendants [7]. Shah et al. [8] reiterated that institutional delivery improves outcomes for expectant mothers as it reduces the risk of maternal mortality. Der et al. [9] also asserted that delivery in a health facility reduces maternal mortality, and availability of emergency obstetric care remains one of the major ways to cut down the maternal mortality rate. Individual factors such as maternal age, parity, level of education, and marital status influence the choice of place of delivery. Other factors include family size and household wealth. The community where a person resides, their socioeconomic status as well as the community’s health infrastructure also influence the decision to deliver at home or at a health institution.

Over the past 5 years, the Northern Region of Ghana has been the only region in the country with coverage below 50%, that is, health facilities are attended by less than 50% of pregnant women according to a Multi-Indicator Survey (MIS) [10]. According to the Ghana Demographic and Health Survey (GDHS) [11], among women who had live births in the Northern Region, 92% received some form of antenatal care (ANC) at a health facility, however only 36.4% of the deliveries were by a skilled provider, which was the lowest among all regions of Ghana. In Ghana even though three quarters of all maternal deaths occur during birth and the immediate postpartum period, skilled birth attendance remains low, and a significant equity gap exists between urban and rural settlements [12, 13]. This study sought to investigate factors that influence the choice of place of delivery among expectant mothers in rural and urban settings of the Northern Region of Ghana.

## Methods

Thaddeus and Maine [14] focused on factors that affect the interval between the onset of obstetric complications and their outcome noting that the latter is mainly affected by delayed treatment which has been shown to be more common with home delivery compared to health-institutional delivery. Their conceptual framework views delay in this context as having three phases namely delay in deciding to seek care, delay in reaching an adequate healthcare facility and thirdly, delay in receiving appropriate care at a suitable healthcare facility. Thematic areas of their conceptual framework included socioeconomic and cultural factors, accessibility of health facilities, quality of the available healthcare and availability of skilled personnel. With this framework as a guide, researchers in this study investigated the factors that hinder skilled delivery in order to explain why expectant mothers may mor may not seek institutional delivery. A mixed-method paradigm, with a predominant focus on quantitative data was used.

Quantitative data collection was conducted to find out the reasons behind the place of delivery choice of the expectant mothers. The quantitative research questions sought to identify the relationship between independent and dependent variables among expectant mothers at the study site. Quantitative data were collected via a representative survey using a structured questionnaire (Additional file 1). A total of 552 women were surveyed: 276 each from the rural and urban study sites. The women were selected based on a two-stage sampling technique. The first phase looked at households selected out of clusters/blocks of individuals whiles the second phase were individual participants from the households/blocks who responded to items on the survey questionnaire. The survey instruments used for data collection were modified versions adopted from GDHS [13]. Participants’ socio-demographic information including marital status, socioeconomic status, age, and educational level were recorded.

Qualitative data were obtained from interviews with selected health workers and a focus group discussion with randomly selected pregnant women and lactating mothers, aged between 15 and 49 years, who volunteered. The interview guide developed for this study is provided as Additional file 2. The interviews were with health workers who work closely with expectant mothers such as community health nurses, midwives, and general nurses. For the interviews, purposive sampling was used to select 9 health professionals from a pool of over 35 staff with a minimum of 5 years of working experience, under the reasoning that in 5 years, the health professional might have been in touch with clients who have provided reasons they chose to deliver at home or at a health facility.

The focus group discussion was used to explore significant quantitative results by probing aspects of the factors influencing the women’s place of delivery choice in rural (Tolon District) and urban (Tamale) settings in northern Ghana. Eight participants each were selected for focus group discussions from the two districts based on the inclusion criteria. The interviews took 15 minutes, and the focus group discussions took 45–60 minutes. The focus group discussion guide is attached as Additional file 3.

The interview and focus group discussion data were analyzed using NVivo version 11 through transcribing, finding emerging themes, coding, and making connections to the research questions. Quantitative data were analyzed using Statistical Package for Social Sciences (SPSS) version 22. Univariate, bivariate, and multivariate analyses were performed. Bivariate analysis established an association between the variables collected. Cross tabulation and stepwise logistic regression were done to assess the predictors of skilled and some determinants of health facility delivery. A regression analysis was performed to determine factors that were significant in affecting the choice of place of delivery among pregnant women within the study area. The study employed indicators such as respondent’s education, the location of respondents, availability of health facility, minimum distance traveled in accessing health care, experience from previous deliveries, and the frequency of attending ANC and seeking of husband’s consent. Variables whose *p*-value were smaller than 0.05 were significant factors influencing the choice of place of delivery among pregnant women.

Ethical clearance was obtained from the Ethics Committee of Tamale Teaching Hospital (TTH), the Tamale Metropolitan Health Directorate as well as the Tolon District Health Directorate (TDHD), and final approval from the Walden University Institutional Review Board (10–02–18-0402836). This study was performed in accordance with the ethical standards laid down in the 2000 Declaration of Helsinki as well as the Declaration of Istanbul 2008. All participants gave their informed consent prior to their inclusion in the study. Written informed consent was obtained from parents of participants under 18 years. Any details that could potentially disclose the identity of any subject(s) in the study was omitted. Data collected for this study was kept as a password protected document with access limited to the principal investigator and research authors only.

## Results

### Qualitative data

Nine health professionals comprised of Midwifes, Physician assistants, and a Clinical nurse were interviewed, 77.8% (*n* = 7) of whom were women. These health professionals had an average of 7 years working experience. Three themes were generated from the interview data namely, Service Provided, Preferred place of delivery, and Factors accounting for Hospital delivery.

#### Service provided by health facility

Respondents were initially asked about the exact services they provide to pregnant women. The feedback from participants revealed that health personnel provide services such as obstetric ultrasonography, clinical examination (palpation and auscultation of fetal heartbeat), preparation of patients for emergency surgery and family planning. Health workers also undertake outreach programs to areas with limited access to health facilities on a regular basis.

#### Preferred place of delivery

The preferred place of delivery was a theme extensively discussed by participants in the two focus groups. Feedback indicated that two options were available to expectant mothers, either delivery at a health facility or at home with the assistance of a traditional birth attendant. Majority of the participants from Tamale (urban area) emphatically indicated that most pregnant women prefer to deliver at health facilities instead of their homes. On the contrary, participants from Tolon District (rural area) indicated that most pregnant women from their district preferred home delivery to delivery at a health facility.

To better understand the variation among women concerning preferred place of delivery, participants were asked for their views on factors accounting for preference for home delivery. The study identified a plethora of factors accounting for pregnant women preferring home delivery. It was observed that there was a traditional notion that a woman’s ability to deliver at home is a sign of strength and faithfulness to her husband. Additionally, participants indicated that some patients believe in the maternal prowess of traditional birth attendants and the behavior of some hospital staff serves as a disincentive to pregnant women to visit health facilities when in labor. Finally, socioeconomic, and environmental factors including poverty as well as poor road networks connecting communities to health centers also play an important but overlooked role in deciding the choice of place of delivery by pregnant women.

#### Factors accounting for hospital delivery

Factors accounting for health facilities as preferred place of delivery as discussed by the participants were:

1. Education of pregnant women by health professionals (enlightenment).
2. Dangers associated with delivering at home.
3. Increase in approval rate of husbands to allow their wives to go through labor at health facilities.
4. Success stories of past deliveries at health facilities.
5. Extent of inhumane treatment given at home during childbirth.

### Quantitative data

**Fig. 1:**
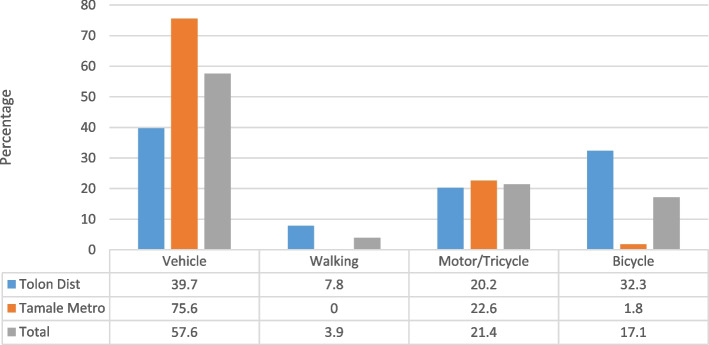
Means of transport for accessing health care

**Fig. 2:**
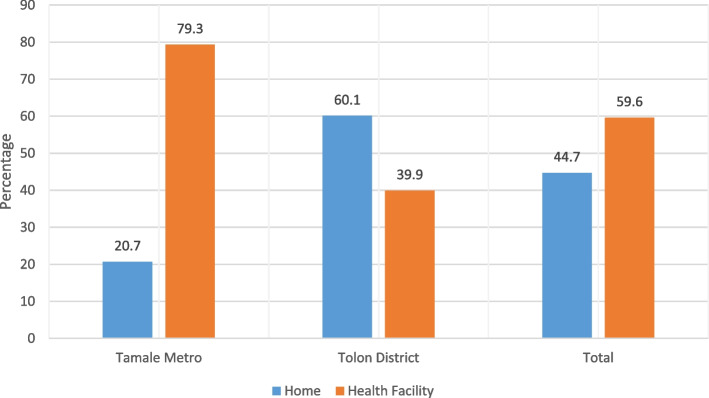
Preferred place of delivery

A total of 552 women participated in the survey among whom 94.7% (*n* = 523) were married (Table 1). In all, 94.7% of the respondents could identify a health facility within their locality (Table 2). More respondents (99.3%) from Tamale answered in the affirmative of a “yes” response. It was also identified that 95.1% of health facilities had the appropriate equipment for delivery (Table 3). With regards to means of transportation, responses were categorized into use of a vehicle, walking, and tricycle/motorcycle use. Vehicular use (57.6%) constituted the dominant means of transportation for accessing healthcare and delivery services for pregnant women followed by motorcycle/tricycle use (21.4%). We also found that 75.6% of the respondents in Tamale used vehicular transportation with a further 22.6% relying on motorcycle/tricycle use (Fig. 1). The findings also showed that 20.7% of the respondents in Tamale preferred home delivery compared to 60.1% of respondents in Tolon District who indicated their readiness to deliver at home (Fig. 2). Statistically significant variables affecting the choice of place of delivery among study participants were found to be educational background, the experience of previous deliveries, the attitude of hospital staff toward pregnant women during labor, and frequency of accessing antenatal care (Table 4).

**Table 1:** Socio-demographic characteristics of the study respondents

Characteristics	Frequency (*n* = 552)	%
Age group (years)		
15–19	22	4.0
20–24	95	17.2
25–29	117	21.2
30–34	141	25.5
35–39	61	11.1
40–44	68	12.3
45–49	48	8.7
Respondent’s educational level		
No formal education	262	47.5
Primary	63	11.4
JHS/middle school	94	17.0
SHS/vocational/technical	88	15.9
Tertiary	45	8.2
Marital status		
Single	7	1.3
Married	523	94.7
Divorced/separated	14	2.5
Widowed	8	1.4
Ethnic background		
Gonja	51	9.2
Dagomba	484	87.7
Others	17	3.1
Household head		
Self	12	2.2
Husband	257	46.6
Father	106	19.2
In-Law	172	31.2
Participant’s religion		
Christianity	44	8.0
Islam	496	89.9
Traditional	12	2.2
Husband’s educational level		
No formal education	283	51.3
Primary	46	8.3
JHS/middle school	74	13.4
SHS/vocational/technical	58	10.5
Tertiary	86	15.6

**Table 2:** Availability of health facilities in study community

	Availability of health facility		Total (%)
	Yes (%)	No (%)	
Tolon District	90.2	9.8	100.0
Tamale	99.3	0.7	100.0
All participants	94.7	5.3	100.0

**Table 3:** Availability of health equipment for delivery

	Availability of health equipment for delivery		Total (%)
	Yes (%)	No (%)	
Tolon District	90.6	9.4	100.0
Tamale	99.6	0.4	100.0
All participants	95.1	4.9	100.0

**Table 4:** Significant factors affecting the choice of place of birth among pregnant women

	*OR*	*SE*	*p*
Respondent’s education	4.990	0.164	0.026
Location of respondent	0.459	0.511	0.498
Availability of health facility	1.813	1.151	0.178
Distance travel to access health facility	1.116	0.187	0.291
Experience of previous deliveries	7.508	0.249	0.006
The attitude of the hospital staff	4.599	0.370	0.032
The frequency of attending ANC	8.237	0.087	0.004
Approval by husband	3.233	1.477	0.490

## Discussion

### Service provided by health facility

The results indicated that health personnel in both locations provided similar services with slight variations in approach. Midwives were the most common providers for ANC and postnatal care in both study areas, but the clinical nurses and physician assistants play greater roles in rural areas. Tran et al. [15] indicated that physician assistants provide ANC and delivery care for more than 25% of pregnant women in rural areas compared to 0.5% in urban areas. ANC counseling among rural women was only given to one fourth of that for urban women [15]. Low household economic status in rural areas might be a barrier preventing women from obtaining expensive services for example laboratory tests and obstetric ultrasonography [16].

### Preferred place of delivery

Participants indicated that most pregnant women prefer to deliver at health facilities instead of delivering at home. However, a segment of pregnant women in Tolon District preferred home delivery (Fig. 2). This finding could be due to unavailability of specialized skills and expertise in rural areas compared to urban areas. A woman’s place of residence, whether rural or urban, could affect the place of delivery [2]. The findings of this study are consistent with the published reports of GDHS and Envuladu et al., [17] where most participants in a rural area preferred home delivery to delivering in a health facility.

### Distribution of place of delivery

Our results indicate a much higher preference for home delivery among rural dwellers vis-a-vis delivery at a health facility as compared to urban dwellers. These findings are consistent with the results of a study in some rural areas conducted by Ravi and Kulasekaran [18] in Tamil Nadu, India, where 69% of the study participants delivered at home. Similar findings were also observed by Tsinuel et al. [19] in a study conducted in Jimma Zone, Ethiopia, where 71% of the women delivered at home. Mukhtar et al. [20] found that 72.6% of women gave birth at home in the tribal areas of district Srinagar.

### Availability of health facilities and place of delivery

The variation between the two districts in terms of the availability of health facilities was not surprising because most developmental projects in Ghana are carried out in urban areas to the detriment of rural areas. The availability of a health facility was not a statistically significant factor that affects the choice of place of delivery in either the rural or urban area in this study. Highly valued virtues like support, care, and companionship were mostly missing in the experience of many women who delivered at health centers. Women who give birth at home do so because they are assured of these virtues when they deliver at home [12].

### Distance and place of delivery

There was no statistically significant association between travel distance and choice of place of delivery. This was consistent with a study by Kifle et al., [21] which indicated that distance to a health facility was not an influential factor to the choice of place of delivery among mothers living in rural communities of Eritrea. However, the findings of this study were contradictory to findings by Abdulmageed and Elnimeiri, [22] where the study noted that distance to a health facility was a significant factor to the choice of place of delivery among pregnant women.

### Implication of social change

This study provides valuable information that can help shape the choices many women make because of how they perceive birth and the cultural traditions as well as taboos surrounding it. This study also provides insight into the importance of an individual’s previous experience with pregnancy, and how that influences how they assess the risks associated with pregnancy. This study also potentially uncovered women’s attitudes to danger linked to childbirth and how they confront that or rationalize the way they deal with it, as well as the norms and values within certain traditions that inform people about the need for and importance of exploring the use of maternal health facilities.

## Conclusion

The choice for a place of delivery by pregnant women may differ in rural and urban areas. Majority of rural women prefer home delivery to facility delivery which is the opposite of the trend observed among urban women. This may be due to several factors that in one way or another influence an expectant mother’s choice of place of delivery. With the high achievement of health facility delivery in urban areas and moderate achievement in rural areas, more must be done to commend and motivate healthcare professionals to maintain the good work in the various health facilities across the country. Education on maternal health services should also be intensified among women with or without formal education.

## Supplementary Information

**Additional file 1.** Questionnaire. Questionnaire with full list of questions that were posed to study participants.

**Additional file 2.** Interview guide. These were questions to the health professionals that sought to find out about the services provided, and attendances of ANC by pregnant women.

**Additional file 3.** Focus Group discussion Guide. These were questions to mothers on their choices, and reasons for their choices.

## Data Availability

The datasets used and/or analyzed during the current study are available from the corresponding author on reasonable request.

## Abbreviations

ANC: Antenatal Care
GDHS: Ghana Demographic and Health Survey
MDGs: Millennium Development Goals
SDGs: Sustainable Development Goals
SPSS: Statistical Package for Social Sciences
WHO: World Health Organization
UNICEF: United Nations Children’s Fund
UNFPA: United Nations Population Fund
MM: Maternal mortality rate
MIS: Multi-indicator Survey
TTH: Tamale Teaching Hospital
TDHD: Tolon District Health Directorate

## References

[1] WHO U, UNFPA WB (2015). Trends in maternal mortality: 1990 to 2015.

[2] Enuameh YA, Okawa S, Asante KP, Kikuchi K, Mahama E, Ansah E, et al. Factors influencing health facility delivery in predominantly rural communities across the three ecological zones in Ghana: a cross-sectional study. PloS one. 2016;11(3):e0152235. 10.1371/journal.pone.0152235 PMC4816577 27031301

[3] Ghana statistical service (GSS), Ghana health service (GHS), and ICF macro-Accra: Ghana Demographic Health Survey 2009. 2008:79–96.

[4] Alkema L (2016). Global, regional, and national levels and trends in maternal mortality between 1990 and 2015, with scenario-based projections to 2030: a systematic analysis by the UN maternal mortality estimation inter-agency group. Lancet.

[5] Organization, W.H (2019). Trends in maternal mortality 2000 to 2017: estimates by WHO, UNICEF, UNFPA, World Bank Group and the United Nations Population Division.

[6] Birmeta K, Dibaba Y, Woldeyohannes D (2013). Determinants of maternal health care utilization in Holeta town, Central Ethiopia. BMC Health Serv Res.

[7] Mills S, Bertrand JT (2005). Use of health professionals for obstetric care in northern Ghana. Stud Fam Plan.

[8] Shah R, Rehfuess EA, Maskey MK, Fischer R, Bhandari PB, Delius M (2015). Factors affecting institutional delivery in rural Chitwan district of Nepal: a community-based cross-sectional study. BMC Pregnancy Childbirth.

[9] Der EM, Moyer C, Gyasi RK, Akosa AB, Tettey Y, Akakpo PK, Blankson A, Anim JT (2013). Pregnancy related causes of deaths in Ghana: a 5-year retrospective study. Ghana Med J.

[10] Ghana. Statistical Service. Multiple Indicator cluster survey, 2011: monitoring the situation of children, women, and men; with an enhanced malaria module and biomarker. Ghana Statistical Service; 2011. https://www.dhsprogram.com/publications/publication-FR262-Other-Final-Reports.cfm.

[11] G. H. S (2015). Ghana Statistical Service , ICF International.

[12] Akum FA (2013). A qualitative study on factors contributing to low institutional child delivery rates in northern Ghana: the case of Bawku municipality. J Community Med Health Educ.

[13] Ghana Statistical Service, Ghana Health Service, and ICF Macro (2014). Ghana demographic health survey.

[14] Thaddeus S, Maine D (1994). Too far to walk: maternal mortality in context. Soc Sci Med.

[15] Tran TK, Gottvall K, Nguyen HD, Ascher H, Petzold M (2012). Factors associated with antenatal care adequacy in rural and urban contexts-results from two health and demographic surveillance sites in Vietnam. BMC Health Serv Res.

[16] Gifford BD (2001). Quality care in a medicaid managed care program: adequacy of prenatal care for teens in Chicago. Public Health Nurs.

[17] Envuladu EA, Agbo HA, Lassa S, Kigbu JH, Zoakah AI (2013). Factors determining the choice of a place of delivery among pregnant women in Russia village of Jos north, Nigeria: achieving the MDGs 4 and 5. Int J Med Biomed Res.

[18] Ravi RP, Kulasekaran RA (2014). Does socio-demographic factors influence women’s choice of place of delivery in rural areas of Tamilnadu state in India. Am J Public Health Res.

[19] Girma T (2008). Traditional newborn care in Jimma town, Southwest Ethiopia. Ethiop J Health Sci.

[20] Mukhtar M, Nelofar M, Quansar R, Khan S, Bashir H (2018). Factors influencing the choice of place of delivery among recently delivered women in tribal areas of district Srinagar: a cross-sectional study. J Med Sci Clin Res.

[21] Kifle MM, Kesete HF, Gaim HT, Angosom GS, Araya MB (2018). Health facility or home delivery? Factors influencing the choice of delivery place among mothers living in rural communities of Eritrea. J Health Popul Nutr.

[22] Abdulmageed SS, Elnimeiri MK (2018). Sociocultural determinants of place of birth among Sudanese women. Int J Commun Med Public Health.

