# Supplementary material for: Diversities in the place of delivery choice: a study among expectant mothers in Ghana
Source: BMC Pregnancy Childbirth. 2022 Nov 25;22:875. doi: 10.1186/s12884-022-05158-0 (PMC9700980; doi:10.1186/s12884-022-05158-0)
Supplement: Supplementary file 1 — Additional file 1. Questionnaire. Questionnaire with full list of questions that were posed to study participants. [file 12884_2022_5158_MOESM1_ESM.docx]

**Questionnaire**

**Instructions:** Please tick or insert the appropriate feedback

**Section A: Socio-Demographic Characteristics of the Mother**

1. Age -------------------------

2. Level of education: None [ ] Primary [ ] JHS [ ] SHS/Voc/ Tech [ ] Tertiary [ ]

3. Type of residence: Compound house [ ] Semi-detached house [ ] Detached house [ ]

4. Residential status: Own residence [ ] Rented house [ ] Family house [ ] others (Specify)............

5. Household head: Self [ ] Husband [ ] Father [ ] In-law [ ]

6. Number of households living in the house ------------------------

7. Ethnicity: Gonja [ ] Dagomba [ ] others (Specify)...................

8. Religion: Christianity [ ] Islam [ ] Traditional [ ] Others (Specify)....................

9. Marital Status: Single [ ] Married [ ] Divorced/Separated [ ] Widowed [ ] Cohabitating [ ]

10. Husband’s educational level: None [ ] Primary [ ] JHS/Middle [ ] SHS/Voc/ Tech [ ] Tertiary [ ]

11. Occupation of husband: Farming [ ] Trading [ ] Public servant [ ] Others (Specify)..............

**Section B: Skilled Deliveries Coverage**

12. Is there a health facility in this community? Yes [ ] No [ ]

13. How many miles will you approximately travel to access health care services? -------------

14. By what means do you travel to the place of health care service? Foot [ ] bicycle [ ] motorbike [ ] passenger car [ ] others (Specify)

15. How many children have you given birth to? ………………..

16. How many of them did you deliver in a health facility?.................

17. How many did you deliver at home?..............

18. Where do you intend to deliver in your current pregnancy? Home [ ] Health Facility [ ] Don’t know [ ]

19. Where did you deliver your last child? Home [ ] Health facility [ ]

20. Was your child weighed at birth? Yes [ ] No [ ]

21. What was your child’s birth weight?................

22. Why do you think some expectant mothers deliver at home instead of a health facility? No health facility [ ] Financial constraints [ ] Attitude of midwives [ ] TBAs are available [ ]

23. Ability to discuss your preferred delivering position with midwives? Very well [ ] Not well [ ] Poorly [ ]

24. Is there privacy in the labor wards? Good privacy [ ] No privacy [ ] Averagely [ ]

25. What was the attitude of the staff towards you? Satisfactory [ ] Unsatisfactory [ ] Bad [ ]

**Section C: Economic Status, Education Status and Level of Empowerment**

26. How many rooms are available for use only by this household? ----------------

27. What type of fuel do you use in cooking? Charcoal [ ] Firewood [ ] Gas [ ] Others (Specify)............

28. Is there electricity in the house? Yes [ ] No [ ]

29. Do you read a newspaper at least once a week? Yes [ ] No [ ]

30. Do you listen to the radio? Almost every day [ ] At least once a week [ ] Less than once a week [ ] Not at all [ ]

31. Do you watch television? Almost every day [ ] At least once a week [ ] Less than once a week [ ] Not [ ]

32. Which of the following do you own? Radio [ ] television [ ]

33. What is your occupation? Unemployed [ ] Trader [ ] Farmer [ ] Civil servant [ ] Others (Specify)

34. Which household resources do you control? Financial [ ] Food materials [ ] Both [ ]

35. Do you decide with your husband on how to spend his income? Yes [ ] No [ ]

36. Do you have the freedom to move or travel? Yes [ ] No [ ]

37. Do you earn more than your partner? Yes [ ] No [ ] Can't tell [ ]

38. Do you take part in decision making on household matters? No [ ] Yes [ ]

If yes

39. Which household matters do you decide together with your partner? Own Health care [ ] Large household purchases [ ] Daily purchase [ ] Children’s education [ ] Household expenditure [ ]

40. Do you have the right to decide how to spend your own money? Yes [ ] No [ ]

41. Who makes the decision if you need to buy clothes for you and the family? Your Husband/partner [ ] Both husband and wife [ ] wife [ ]

42. Who makes the decision if you need to buy large household items/furniture? Your Husband/partner [ ] Both husband and wife [ ] Wife [ ]

43. Who makes the decision whether a child is sick enough to go for treatment? Your Husband/partner [ ] Both husband and wife [ ] Wife [ ]

44. Who makes the decision whether you should work outside of the home? Your Husband/partner [ ] Both husband and wife [ ] Wife [ ]

45. Who makes the decision when your children have stationeries /school needs to be addressed? Your Husband/partner [ ] Both husband and wife [ ] Wife [ ]

46. Who makes the decision about how to spend the families income? Your Husband/partner [ ] Both husband and wife [ ] Wife [ ]

47. What is your family’s main source of income? Husband’s earnings [ ] Own earnings [ ] Yours and husbands earnings [ ] Others (Specify)........................

48. How often do you have money that you alone can decide how to spend? Always [ ] Often

[ ] Sometimes [ ] Never [ ]

49. Do you currently have any type of saving Scheme? Yes, partners saving [ ] Yes, mine and partners saving [ ] Yes, self-saving [ ] No, we don’t have any saving [ ]

50. Do you attend antenatal clinics when pregnant? yes [ ] No [ ]

51. How many times did you attend ANC in during your last pregnancy? …………….

52. Who decides when you should go for ANC? My husband [ ] Myself [ ] In laws [ ]

53. Who gives you money to go for ANC? Self [ ] Husband [ ] In laws [ ]

54. Are there midwives in the closest or nearest health facility? Yes [ ] No [ ]

55. Does your husband approve of facility delivery? Yes { ] No [ ]

56. Is there equipment to conduct deliveries in the nearest health facility? Yes [ ] No [ ]
