# Supplementary material for: Diversities in the place of delivery choice: a study among expectant mothers in Ghana
Source: BMC Pregnancy Childbirth. 2022 Nov 25;22:875. doi: 10.1186/s12884-022-05158-0 (PMC9700980; doi:10.1186/s12884-022-05158-0)
Supplement: Supplementary file 2 — Additional file 2. Interview guide. These were questions to the health professionals that sought to find out about the services provided, and attendances of ANC by pregnant women. [file 12884_2022_5158_MOESM2_ESM.docx]

**Title of Study: Diversities in the Place of Delivery Choice: A study among expectant Mothers in Ghana**

**Semi structured Interview Questions**

**Semi-structured interview questions with the Health Professionals**

Researcher's questions

1. What are the specific maternal health services that you provide to pregnant women in this facility?
2. In your opinion, where do most pregnant women usually go to give birth?
3. If in your opinion most pregnant women prefer to give birth at home then what could be the reasons?
4. If in your opinion most pregnant women prefer to deliver at health facilities then what could be the possible causes, and if they do not prefer health facility delivery, why not?
5. How would you describe the attendance of ANC by pregnant women of this community?
