# Supplementary material for: Diversities in the place of delivery choice: a study among expectant mothers in Ghana
Source: BMC Pregnancy Childbirth. 2022 Nov 25;22:875. doi: 10.1186/s12884-022-05158-0 (PMC9700980; doi:10.1186/s12884-022-05158-0)
Supplement: Supplementary file 3 — Additional file 3. Focus Group discussion Guide. These were questions to mothers on their choices, and reasons for their choices. [file 12884_2022_5158_MOESM3_ESM.docx]

**Title of Study: Diversities in the Place of Delivery Choice: A study among expectant Mothers in Ghana**

**Focus Group Discussion Guide**

**Focus Group discussion Guide for Expectant and lactating mothers (Who meet the inclusion criteria)**

**1.** Where do you often go for maternal health services when pregnant and why?

**2.** Where do you choose to go for childbirth when in labor and why?

3. Whom do you prefer to conduct your delivery and why?

4. If you prefer informal providers like TBAs (Traditional Birth Attendants), then, why? What informs your decision to decide to give birth at the facility of a TBA?

5. If you prefer other than TBA, then why?
